# Multi-view fusion based on graph convolutional network with attention mechanism for predicting miRNA related to drugs

**DOI:** 10.1371/journal.pcbi.1013703

**Published:** 2025-11-14

**Authors:** Nan Sheng, Yunzhi Liu, Ling Gao, Lei Wang, Lan Huang, Yan Wang

**Affiliations:** 1 College of Computer Science and Technology, Jilin University, Changchun, China; 2 Key Laboratory of Symbolic Computation and Knowledge Engineering of the Ministry of Education, Jilin University, Changchun, China; Christian Albrechts Universitat zu Kiel, GERMANY

## Abstract

MicroRNAs (miRNAs) play crucial roles in cancer progression, invasion, and response to treatment, particularly in regulating anticancer drug resistance and sensitivity. Identifying potential human miRNA-drug associations (MDAs) that manifest as resistance or sensitivity relationships offers valuable insights for cancer treatment and drug development. With the growing availability of biological data, computational methods have emerged as powerful tools to complement experimental approaches. However, limited attention has been paid to computational prediction of MDAs. Furthermore, existing approaches typically rely on known MDA information, overlooking the valuable insights available from multi-source data related to miRNAs and drugs. In this study, we present a multi-view fusion-based graph convolutional network with attention mechanism (MGCNA) to predict miRNA-associated drug resistance/sensitivity. Specifically, MGCNA integrates macro- and micro- level information of miRNAs and drugs to construct multi-view node features from different perspectives. The proposed multi-view graph convolutional network (GCN) encoder obtains miRNA and disease features from different views and learns adaptive importance weights of the embedding using an attention mechanism. Extensive experiments on manually curated benchmark datasets demonstrate that MGCNA outperforms existing baseline methods. Case studies of two common drugs further establish MGCNA’s effectiveness in discovering novel MDAs.

## Author summary

Cancer treatments often fail because tumors develop resistance to drugs, or conversely, become unexpectedly sensitive, and microRNAs (miRNAs) play a key role in this process. While discovering miRNA-drug relationships can improve cancer therapy, experimental methods alone are time-consuming and costly. Computational predictions could accelerate this process, but most current methods rely too much on existing data and miss hidden patterns in broader biological information. We developed a new deep learning model, MGCNA, to predict miRNA-drug resistance/sensitivity. Our approach combines multiple types of biological data and automatically learns which features matter most, improving accuracy over existing methods. When tested on real-world datasets, MGCNA outperformed other models, and we confirmed its predictions with case studies on common anticancer drugs (Docetaxel and Sorafenib). This tool could help researchers develop more effective cancer treatments by identifying which miRNAs might make specific drugs work better. We’ve made all our data and code freely available to help other scientists build on our work and further advance cancer therapy research.

## Introduction

Cancer is the leading cause of death worldwide, posing a significant threat to human health and survival [1]. Chemotherapy remains a cornerstone of cancer treatment. However, the emergence of drug resistance represents a critical challenge in cancer treatment, hindering therapeutic efficacy and resulting in poor clinical prognosis [2]. miRNAs are single-stranded non-coding RNAs (ncRNAs) approximately 22 nucleotides in length that play crucial regulatory roles in various biological processes. Growing evidence indicates that miRNAs are implicated in the pathogenesis of human diseases, and significantly influence drug resistance and sensitivity [3,4]. For instance, Wu et al. demonstrated that miRNA-93-5p could promote gemcitabine resistance in pancreatic cancer cells by targeting the PTEN-mediated PI3K/Akt signaling pathway [5]. In cholangiocarcinoma treatment, 5-fluorouracil (5-FU) serves as a common drug. Du et al. discovered that the lncRNA FALEC enhances drug resistance by competitively regulating the miR-20a-5p/SHOC2 axis [6]. Moreover, miRNAs can influence not only the development of drug resistance during treatment but also promote drug sensitivity. For example, Niu et al. revealed that miR-195 enhanced cisplatin sensitivity in oral cancer cells through downregulation of MAPK kinase 1 expression [7]. Additionally, miR-885-5p increased the sensitivity of cholangiocarcinoma cells to 5-FU and improved therapeutic efficacy by targeting MTPN [8]. Understanding these miRNA-drug interactions is therefore crucial for treatment optimization and drug development.

Due to the crucial role of miRNAs in regulating drug resistance and sensitivity, several miRNA-drug association databases have been developed in recent years, including NoncoRNA [9], ncDR [10], and ncRNADrug [11]. Traditional laboratory experiments to identify these potential associations are time-consuming and costly. As a result, computational methods have emerged as a promising alternative benefiting from the development of these data resources. For example, Huang et al. developed an end-to-end GCN that integrates miRNA expression profiles and drug fingerprinting information to learn potential embeddings and predict miRNA-associated drug resistance [12]. Deng et al. constructed a miRNA-drug bipartite network using known MDAs and applied a dual-heterogeneous graph representation learning method to predict the association scores [13]. Li et al. first constructed an ncRNA-drug resistance bipartite graph, which does not distinguish ncRNA node types [14]. Subsequently, linear residual graph convolution was employed to extract node embeddings from the network and infer potential scores. Similarly, Zhang et al. built an ncRNA-drug bipartite graph and incorporated LightGCN with self-supervised learning to learn node features [15]. Wei et al. developed a miRNA-drug bipartite graph, combining topological and feature contrastive learning with collaborative filtering algorithms to infer potential miRNA-drug sensitivity [16]. Recently, Zhou et al. integrated graph autoencoder and random path masking techniques to predict potential miRNA-drug resistance [17]. Although aforementioned methods have achieved promising results in miRNA-drug resistance and sensitivity prediction, they usually suffer from one or two following limitations: (1) The annotated miRNA-drug association data remain limited, resulting in sparse and noisy bipartite graphs. This sparsity particularly affects graph neural network methods, which rely heavily on graph structure to learn node embeddings. (2) Since miRNAs regulate drug efficacy by regulating gene expression, the integration of miRNA-gene and drug-gene associations becomes crucial for predicting the relationship between miRNAs and drugs.

To address the above challenges, we propose a novel multi-view fusion framework based on graph convolutional networks with an attention mechanism (MGCNA). Specifically, we incorporate diverse types of miRNA- and drug-related domain knowledge to construct a multi-view network that characterizes node attributes from multiple perspectives, encompassing miRNA sequences, miRNA-gene interactions, drug structures, drug-gene interactions, and miRNA-drug associations. This approach offers a key advantage, it leverages multi-source data information related to both miRNAs and drugs, while avoiding excessive dependence on known MDAs. For each view, MGCNA learns the node representations in respective view spaces using the multi-view GCN as the backbone. Furthermore, it utilizes an attention mechanism to automatically learn importance weights for different views, enabling adaptive fusion of information. Through this approach, node labeling supervises the learning process and adaptively adjusts the weights to complement view information across dimensions, thereby enhancing prediction performance. The key contributions of this work are as follows:

- We propose a multi-view fusion framework based on graph convolutional network with attention mechanism, called MGCNA, which aims to fuse macro- and micro-level features of miRNAs and drugs to enhance the accuracy of MDA prediction.
- We implement a comprehensive multi-view architecture that characterizes miRNAs and drugs from diverse perspectives using multi-source information. The framework employs a multi-channel GCN encoder to capture view-specific information, and integrates an attention mechanism to adequately fuse information across different views.
- Our extensive experiments on a curated benchmark dataset demonstrate that MGCNA outperforms existing baseline approaches and effectively leverages multi-view information to improve MDA prediction. The case studies further verify that MGCNA can effectively identify novel drug resistance/sensitivity related miRNAs.

## Materials and methods

**Fig 1:**
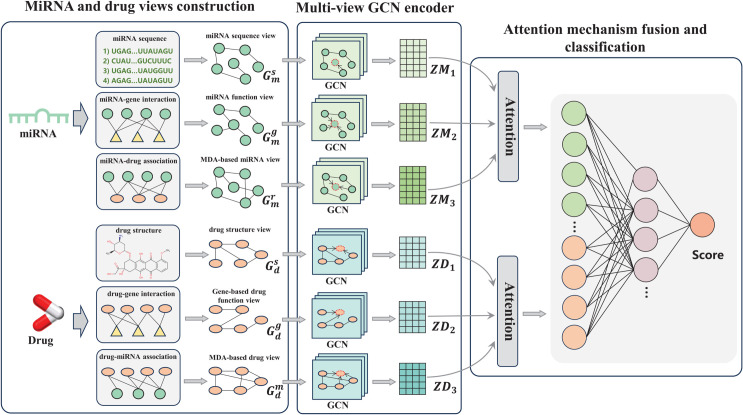
Overview of the proposed MGCNA model.

Fig 1 illustrates an overview of our proposed multi-view fusion framework-based graph convolutional network with attention mechanism (MGCNA). The framework consists of three main components. Firstly, the multi-source domain knowledge of miRNAs and drugs is comprehensively considered from both macro and micro perspectives to construct multiple views. These encompass 6 types of views: miRNA sequence view, miRNA function view, drug structure view, gene-based drug function view, and MDA-based miRNA and drug views. Secondly, a multi-view GCN is constructed as the backbone of the encoder to fully learn the node representation in each view space. Finally, considering that miRNA-drug pair labels may be associated with different views, MGCNA utilizes an attention mechanism to adaptively fuse these views with the learned weights, thereby extracting the most relevant information for final prediction. Our ultimate goal is to predict the likelihood of MDAs, including resistance/sensitivity associations.

### Benchmark datasets

Multi-source data pertaining to miRNA and drug associations were collected from several public biomedical databases to construct comprehensive multi-view networks. (1) Due to the limited availability MDA datasets, we manually established a benchmark dataset derived from the ncRNADrug [11]. This database encompasses manually curated and predicted disease resistance/sensitivity-related ncRNAs (including miRNAs, lncRNAs, and circRNAs). In this paper, we focused on experimentally validated human miRNA-drug resistance and sensitivity associations, excluding lncRNA and circRNA data. Following screening and preprocessing protocols, a total of 8720 association pairs between 1578 miRNAs and 156 drugs were obtained. (2) For drugs and miRNAs, their SMILES strings and miRNA sequences were downloaded from the Drugbank [18] and miRbase [19], respectively. (3) Based on this criterion, we extracted gene relationships associated with miRNAs and drugs from the miRTarBase [20] and Comparative Toxicogenomics Database (CTD) [21], yielding 242924 miRNA-gene associations (MGAs) and 96456 drug-gene associations (DGAs). The statistical information of the dataset is presented in Table 1.

**Table 1:** The statistics for benchmark datasets.

miRNAs	Drugs	Genes	MDAs	MGIs	DGAs
1578	156	14455	8720	242924	96456

### miRNA and drug views construction

In this section, we apply multi-source data related to miRNAs and drugs to calculate similarity view, thereby comprehensively characterizing feature miRNAs and drugs. The detailed computational methods are presented as follows.

#### miRNA sequence view.

Despite miRNA sequences being relatively short (20-24 nucleotides), they contain information for gene expression regulation. The k-mer approach, a widely used RNA sequence descriptor, quantifies the frequency of k consecutive nucleic acids, effectively capturing local sequence features. In this study, we first employed k-mer to extract sequence information from miRNA sequences, which incorporate three types of k-mer features: 1-mer (A, C, T, G), 2-mer (AA, AC, ..., TG), and 3-mer (AAA, AAT, ..., GGG). These features were subsequently concatenated into an 84-dimensional vector. To quantify the sequence similarity between miRNA *m*_*i*_ and *m*_*j*_, cosine similarity was implemented as follows:

$$G_{m}^{s}(i,j)=\frac{x_{i}\cdot x_{j}}{\left|x_{i}\right|\cdot \left|x_{j}\right|}$$ (1)

where *x*_*i*_ and *x*_*j*_ denote the k-mer feature vectors of *m*_*i*_ and *m*_*j*_, respectively. The resulting $G_{m}^{s}\in {ℛ}^{N_{m}\times N_{m}}$ can be regarded as the miRNA sequence view, where *N*_*m*_ indicates the number of miRNAs.

#### miRNA functional view.

During disease treatment, miRNAs can modulate target genes to influence drug resistance and sensitivity. Therefore, we constructed a miRNA functional view $G_{m}^{g}\in {ℛ}^{N_{m}\times N_{m}}$ based on their regulatory relationships with genes. Specifically, based on the assumption that similar miRNAs (genes) interacting with similar genes (miRNAs) will have similar profiles, the Gaussian interaction profile kernel (GIPK) was utilized to compute the miRNA functional similarity. The GIPK similarity between miRNAs *m*_*i*_ and *m*_*j*_ is defined as:

$$G_{m}^{g}(i,j)=\exp\left(-\gamma_{m}{\left\|IP\left(m_{i}\right)-IP\left(m_{j}\right)\right\|}^{2}\right)$$ (2)

where *IP*(*m*_*i*_) and *IP*(*m*_*j*_) represent the *i*-th and *j*-th rows of the miRNA-gene association matrix, respectively. The parameter $\gamma_{m}$ denotes the normalized kernel bandwidth:

$$\gamma_{m}=\gamma_{m}^{\prime }/\left(\frac{1}{N_{m}}\sum_{i=1}^{N_{m}}{\left\|IP\left(m_{i}\right)\right\|}^{2}\right)$$ (3)

where $\gamma_{m}^{\prime }$ is set to 1.

#### Drug structure view.

The Molecular ACCess System (MACCS) fingerprint is a 167-bit molecular representation, with 166 bits encoding the presence or absence of specific molecular substructures. It is one of the most widely implemented structural descriptors for drugs. In our study, MACCS fingerprint features were generated utilizing the RDKit [22] based on the drug SMILES strings. The structural similarity between drugs *d*_*i*_ and *d*_*j*_ was subsequently quantified using the Tanimoto coefficient:

$$G_{d}^{s}(i,j)=\frac{\left|A\left(d_{i}\right)\cap A\left(d_{j}\right)\right|}{\left|A\left(d_{i}\right)\cup A\left(d_{j}\right)\right|}$$ (4)

where *A*(*d*_*i*_) and *A*(*d*_*j*_) denote the sets of MACCS fingerprint features for drugs *d*_*i*_ and *d*_*j*_, respectively. The resulting $G_{d}^{s}\in {ℛ}^{N_{d}\times N_{d}}$ can be considered as the drug structural view, where *N*_*d*_ denotes the number of drugs.

#### Gene-based drug function view.

The gene-based functional drug view $G_{d}^{g}\in {ℛ}^{N_{d}\times N_{d}}$ is similar to the miRNA functional view calculation, which is derived using drug-gene interactions and the GIPK similarity. The similarity between drugs *d*_*i*_ and *d*_*j*_ is expressed as:

$$G_{d}^{g}(i,j)=\exp\left(-\gamma_{d}{\left\|IP\left(d_{i}\right)-IP\left(d_{j}\right)\right\|}^{2}\right)$$ (5)

$$\gamma_{d}=\gamma_{d}^{\prime }/\left(\frac{1}{N_{d}}\sum_{i=1}^{N_{d}}{\left\|IP\left(d_{i}\right)\right\|}^{2}\right)$$ (6)

where *IP*(*d*_*i*_) and *IP*(*d*_*j*_) represent the *i*-th and *j*-th rows of the drug-gene interaction matrix, respectively. The parameter $\gamma_{d}^{\prime }$ is initialized to 1.

#### MDA-based miRNA and drug view.

Known MDA demonstrates direct regulatory relationships between miRNAs and drugs, such as resistance or sensitivity. Leveraging this interaction information is crucial for discovering potential miRNA-drug pairs. Consequently, the MGCNA constructed miRNA view $G_{m}^{r}\in {ℛ}^{N_{m}\times N_{m}}$ and drug view $G_{d}^{m}\in {ℛ}^{N_{d}\times N_{d}}$ based on known MDAs, implementing the aforementioned GIPK similarity calculations.

For the derived miRNA views $\left(G_{m}^{s},G_{m}^{g},G_{m}^{r}\right)$ and drug views $\left(G_{d}^{s},G_{d}^{g},G_{d}^{m}\right)$, this work applied a threshold function to filter out redundant and insignificant node relationships, thereby optimizing information propagation in the graph neural network. The edge reconstruction is defined as:

$$G(i,j)=\left\{ \begin{array}{c} 1,\;\text{if }G(i,j)\geq \theta \\ 0,\;\text{otherwise} \end{array}\right.$$ (7)

where *θ* is a hyperparameter that controls the similarity threshold between nodes.

### Multi-view graph convolutional network encoder

GCNs are efficient and powerful graph neural network architectures capable of aggregating neighborhood information from nodes and capturing complex inter-entity relationships within networks [23]. To thoroughly analyze and integrate structural representations of miRNAs and drugs from multiple perspectives, we apply multi-view GCNs to encode different views. Taking the miRNA view encoding as an example, consider the miRNA view network $G_{m}=(V_{m},G_{m}^{s},G_{m}^{g},G_{m}^{r})$, where $V_{m}$ is the set of nodes with $|V_{m}|=N_{m}$. As graph structure is primarily represented through an adjacency matrix, we reformulate the miRNA view as $G_{m}=(V_{m},A_{m}^{s},A_{m}^{g},A_{m}^{r})$. The network *G*_*m*_ is processed by the multi-view GCN encoding module to extract new miRNA embedding representations. For layer *l*, the propagation rule can be defined as:

$$ZM_{i}^{\left(l\right)}=\sigma \left({\tilde{D}}_{i}^{-\frac{1}{2}}{\tilde{A}}_{i}{\tilde{D}}_{i}^{-\frac{1}{2}}ZM_{i}^{\left(l-1\right)}W_{i}^{\left(l\right)}\right),i=3$$ (8)

where ${\tilde{A}}_{i}=A_{i}=(A_{m}^{s},A_{m}^{g},A_{m}^{r})$, as the diagonal values are set to 1 when constructing the similarity view. ${\tilde{D}}_{ii}=\sum_{j}{\tilde{A}}_{ij}$ and $W_{i}^{\left(l\right)}$ is the trainable weight matrix for the *i*-th view at layer *l*. *σ* is the ReLU activation function. $Z_{i}^{\left(l-1\right)}$ represents the (*l*–1)-th layer embedding. $ZM_{i}^{\left(0\right)}=X_{m}\in {ℛ}^{N_{m}\times h}$, *X*_*m*_ is the randomly initialized miRNA feature matrix, *h* denotes the initialized embedding dimension. The final output of the multi-view GCN for the miRNA view is represented as $\left\{ZM_{1},ZM_{2},ZM_{3}\right\}$.

Similarly, the drug view network $G_{d}=(V_{d},G_{d}^{s},G_{d}^{g},G_{d}^{m})$ is fed into the multi-view GCN encoder module to generate new representation $\left\{ZD_{1},ZD_{2},ZD_{3}\right\}$. The subsequent section will discuss the design of the fusion function for aggregating different view representations to achieve a more comprehensive semantic representation.

### View attention mechanism

For miRNA and drug, different views contain distinct contextual information. MGCNA implements a view attention mechanism to adaptively calculate the attention score of each view and then integrate the more important view features. Taking miRNA view aggregation as an example, for multi-view embeddings $zm_{1}^{m},zm_{2}^{m},zm_{3}^{m}$ of miRNA node *m*, the aggregated representation is defined as:

$$zm^{m}=\sum_{i=1}^{3}\alpha_{i}zm_{i}^{m}$$ (9)

where $\alpha_{i}$ denotes the attention coefficient for each miRNA view embedding, which is calculated as follows:

$$s_{i}^{m}=q^{T}\cdot \tanh\left(W_{a}\cdot {\left(zm_{i}^{m}\right)}^{T}+b_{a}\right)$$ (10)

$$\alpha_{i}=\frac{\exp\left(s_{i}^{m}\right)}{\sum_{j=1}^{3}\exp\left(s_{j}^{m}\right)}$$ (11)

where *W*_*a*_ and *b*_*a*_ are learnable parameters representing the weight matrix and bias vector, respectively. *q* is a learnable query vector. Through the same process, we derive the final embeddings for the drug view *ZD*. These integrated embeddings serve as inputs for the subsequent MDA prediction task.

### Objective function

Based on the fused miRNA embeddings *ZM* and drug embeddings *ZD* through the attention mechanism, we construct miRNA-drug pair embeddings to predict their interaction probabilities. For a given miRNA node *m* and drug node *n*, their interaction probability ${\hat{y}}_{mn}$ is predicted using a multi-relational modeling decoder:

$${\hat{y}}_{mn}=f_{MLP}\left(zm_{m}+zd_{n}\left\|zm_{m}⊙zd_{n}\right\|zm_{m},zd_{n}\right)$$ (12)

where *zm*_*m*_ is the embedding of miRNA node *m* (corresponding to *m*-row of *ZM*), *zd*_*n*_ is the embedding of drug node *n* (corresponding to *n*-row of *ZD*). The operators || and ⊙ denote concatenation and element-wise product, respectively. *f*_*MLP*_ consists of a two-layer fully connected neural network. The model is optimized using cross-entropy loss as follows:

$$L=-\frac{1}{\varphi }\sum_{m,n\in \varphi }y_{mn}\log\left({\hat{y}}_{mn}\right)+\left(1-y_{mn}\right)\log\left(1-{\hat{y}}_{mn}\right)$$ (13)

where φ represents the training set of miRNA-drug pairs, *y*_*mn*_ and ${\hat{y}}_{mn}$ denote the true and predicted labels for the interaction between the *m*-th miRNA and the *n*-th drug, respectively.

## Experiments and results

### Implementation and evaluation metrics

In practice, MGCNA is implemented in Python using the PyTorch deep learning framework and trained using the Adam optimizer with an initial learning rate of 0.0005 and weight decay of 0.0005. The implementation leverages key libraries, including NumPy v1.24.4, PyTorch v2.1.2, PyTorch-Geometric v2.4.0. All experiments are conducted on a system equipped with a GeForce RTX 4070 GPU to accelerate model training and inference. Moreover, dropout with a drop rate of 0.5 is applied to prevent overfitting. For the miRNA and drug views, a two-layer GCN is utilized for encoding based on prior experience. The similarity threshold *θ* is searched in [0.5, 0.9]. More detailed settings are available from GitHub: https://github.com/sheng-n/MGCNA.

In this study, 5-fold cross-validation (5-cv) is employed to evaluate the performance of MGCNA and baseline methods for predicting potential drug-related miRNAs. Known MDAs are treated as positive samples, while unknown samples serve as candidate negative samples. To balance the dataset, MGCNA selects negative samples equal in number to the positive samples from the candidate samples. In each fold, the positive and negative samples are randomly partitioned into 5 equal subsets, with 4 used for training and the remaining 1 for testing. This validation process is repeated 5 times, with each subset serving as the test set once. To prevent data leakage, the MDA-based miRNA and drug view is recomputed and reconstructed during each fold. We adopt two commonly used evaluation metrics, the area under the receiver operating characteristic curve (AUC) and the area under the precision-recall curve (AUPR), for measuring model performance.

### Analysis of multiple views

In this study, MGCNA primarily integrates miRNA and drug multi-view information to predict MDAs. To demonstrate the effectiveness of multi-view learning, we tested MGCNA with various view combinations and conducted ablation experiments by removing specific views. As shown in Table 2, we have the following observations: (1) MGCNA achieves best performance (AUC=0.9533, AUPR=0.9513) when combining miRNA views ($G_{m}^{s}$, $G_{m}^{r}$), and drug views ($G_{d}^{s}$, $G_{d}^{g}$, $G_{d}^{m}$). One possibility is that incorporating excessive miRNA information from miRNA-gene interactions introduces noise, which slightly reduces overall performance. (2) Removing the miRNA view $G_{m}^{r}$ and drug view $G_{d}^{m}$ significantly decreases MGCNA’s performance, highlighting the importance of known miRNA-drug relationships for predicting unknown MDAs. (3) When utilizing only gene-based miRNA and drug functional views $G_{m}^{g}$ and $G_{d}^{g}$, MGCNA achieved the lowest performance (AUC = 0.7795, AUPR = 0.8028), indicating that gene features alone provide limited prediction capability. (4) Incorporating domain knowledge of miRNA sequences, drug structures, and drug-related genes, enhances prediction accuracy. Overall, MGCNA performs best when integrating most available views, demonstrating robustness even when certain views are missing. This flexibility is a key advantage of the model.

**Table 2:** Performance comparison of MGCNA on multiple views. (Bold: best; Underline: runner-up).

$G_{m}^{s}$	$G_{m}^{g}$	$G_{m}^{r}$	$G_{d}^{s}$	$G_{d}^{g}$	$G_{d}^{m}$	AUC	AUPR
✓	✗	✗	✓	✗	✗	0.9275	0.9142
✗	✓	✗	✗	✓	✗	0.7795	0.8028
✗	✗	✓	✗	✗	✓	0.9308	0.9275
✗	✗	✓	✓	✓	✓	0.9384	0.9379
✓	✓	✓	✗	✗	✓	0.9421	0.9394
✗	✓	✓	✗	✓	✓	0.9392	0.9311
✓	✗	✓	✓	✗	✓	0.9425	0.9406
✓	✓	✗	✓	✓	✗	0.9102	0.8898
✗	✓	✓	✓	✓	✓	0.9401	0.9364
✓	✗	✓	✓	✓	✓	**0.9533**	**0.9513**
✓	✓	✗	✓	✓	✓	0.9275	0.9163
✓	✓	✓	✗	✓	✓	0.9396	0.9387
✓	✓	✓	✓	✗	✓	0.9398	0.9388
✓	✓	✓	✓	✓	✗	0.9304	0.9277
✓	✓	✓	✓	✓	✓	0.9486	0.9467

### Comparison with other baseline methods

To validate our proposed methods, we chose the high-performance version of MGCNA, and conducted comparative experiments against several baseline models on the benchmark dataset. This baselines included DAESTB [24], GCNNMMA [25], SubMDTA [26], GraphDTA [27], and ML-DTI [28]. A brief description of baseline methods and their model setting are provided in the S1 Text.

**Fig 2:**
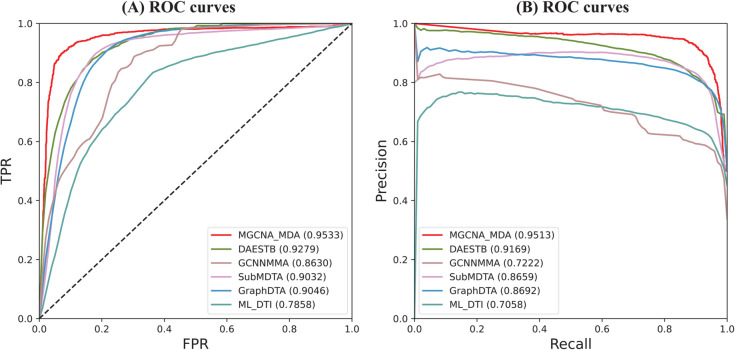
Performances of MGCNA and baselines evaluated by ROC curves and PR curves in benchmark datasets.

To make the comparison as fair as possible, all methods are evaluated using 5-cv for prediction performance assessment. Considering that miRNA sequences are significantly shorter than protein sequences, we fine-tune the competing methods for the miRNA-drug prediction task to optimize their performance. The experimental results are presented in Fig 2. We have the following observations and analysis: (1) MGCNA demonstrates superior performance compared to all baselines, achieving an AUC of 0.9533 and AUPR of 0.9513, establishing the effectiveness of the proposed approach. (2) DAESTB uses an autoencoder to extract embeddings from node-pair features based on multiple miRNA and drug similarities, whereas our direct feature extraction from similarity graphs using graph neural networks proved more effective. (3) MGCNA significantly outperformed GCNNMM, SubMDTA, GraphDTA, and ML-DTI. This performance advantage can be attributed to the integration of multi-source information, which substantially enhances model performance compared to approaches utilizing only drug molecular graph and miRNA sequences. These results indicate that the strategy of fusing multi-view information with an attention mechanism enhances MGCNA’s ability to predict potential miRNA-drug relationships.

### Ablation study

**Fig 3:**
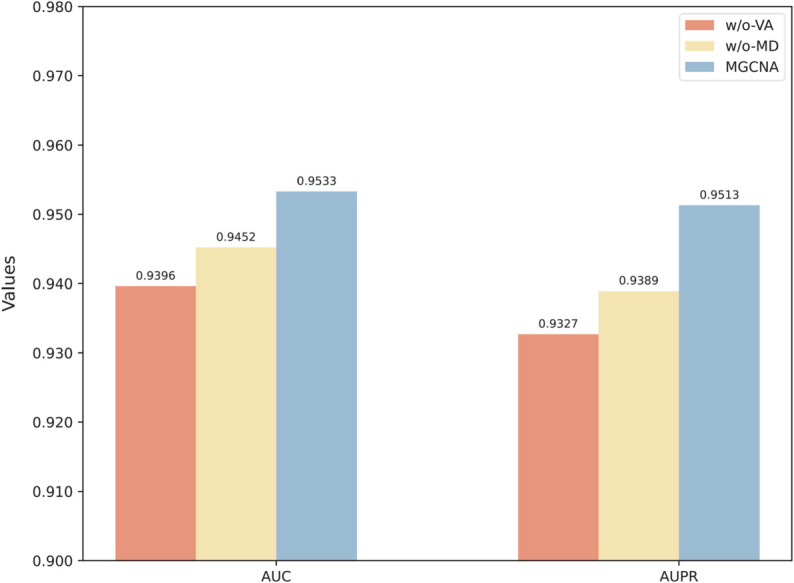
The comparison of MGCNA and its variants.

To investigate the importance of key components: view attention mechanism and multi-relational modeling decoder, we conducted ablation studies using the following MGCNA variants, (1) MGCNA-w/o-VA without view attention, and employs a summation approach to fuse miRNA and drug views. (2) MGCNA-w/o-MD without multi-relational modeling decoder, constructing miRNA-drug node pair embeddings through simple concatenation. From the results in Fig 3, we can draw the following conclusions: (1) MGCNA consistently outperforms all the other variants, with the removal of either component diminishing its prediction capability. (2) MGCNA-w/o-VA and MGCNA-w/o-MD achieve performance drop, demonstrating that both the view attention mechanism and multi-relational modeling decoder are essential components of MGCNA’s architecture.

### Parameter sensitivity study

**Fig 4:**
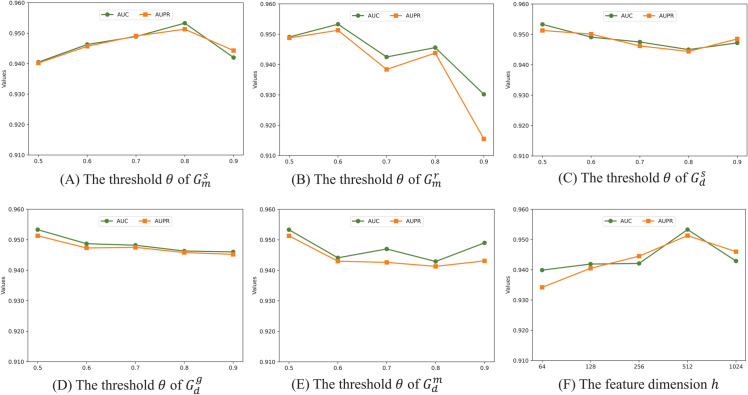
Analysis of the threshold *θ* and initialized feature dimension *h.*

In this section, we systematically analyze the parameter sensitivity of MGCNA, focusing on two key parameters: the similarity threshold *θ* and the initialized node embedding dimension *h*. The experimental results are presented in Fig 4.

**Analysis of threshold *θ***: The threshold *θ* is a crucial parameter controlling the density of miRNA and drug views. We evaluated values ranging from 0.5 to 0.9 across different views. In the miRNA view, the performance initially increases and subsequently decreases as the thresholds increase in views $G_{m}^{s}$ and $G_{m}^{r}$ (Figs 4A and 4B). We vary the value of *θ* in drug views $\left(G_{d}^{s},G_{d}^{g},G_{d}^{m}\right)$, and corresponding results are shown in Figs 4C, 4D, and 4E. With the increase of *θ*, the performance consistently declines, and optimum points for three drug views are at 0.5. All in all, since MGCNA employs computational similarity to construct views, lower *θ* can introduce redundant edges in the view, generating noise that adversely affects model performance. Conversely, when *θ* is too high, it generates sparse views that are insufficient for characterizing the node domain knowledge.

**Analysis of initialized feature dimension *h***: We evaluated the impact of the initial embedding dimension *h*, with it selected from 64, 128, 256, 512, and 1024. For each layer of the GCN, the embedding dimension is set to *h*/2 and *h*/4, respectively. As illustrated in Fig 4F, the performance of MGCNA initially increases and subsequently decreases as *h* increases. This phenomenon may be due to the fact that lower dimensions are not sufficient to represent the node feature, while redundant embeddings are introduced at higher dimensions. Based on these results, the optimal value of *h* is set as 512.

### Performance comparison in cold-start scenarios

To better simulate real-world MDA prediction scenarios, we evaluated MGCNA’s performance under conditions involving unknown drugs and unknown miRNAs. We conducted three cold-start scenarios: (1) miRNA cold-start, where miRNAs in the training set do not appear in the test set, (2) drug cold-start, where drugs in the training set do not appear in the test set, and (3) miRNA-drug pair cold-start, where neither the miRNAs nor the drugs in the test set are present in the training set. Table 3 presents the cold-start results for all methods on the benchmark dataset. From Table 3, several conclusions are obtained as follows: (1) Our model achieves superior overall performance in all cold-start settings, demonstrating that multi-source data fusion enables effective information supplementation and enhances prediction accuracy. (2) All models showed significant performance degradation in the drug cold-start setting, likely due to insufficient drug class representation in our dataset. (3) The miRNA-drug pair cold-start scenario posed the greatest challenge, with all models showing reduced performance. Despite these challenges, MGCNA maintains relatively stable and robust performance in cold-start scenarios, confirming the effectiveness of our multi-view fusion network architecture for discovering novel MDAs.

**Table 3:** Performance comparison of MGCNA and baseline methods under cold-start settings.

Methods	miRNA code-start		Drug code-start		miRNA-drug pair code-start	
	AUC	AUPR	AUC	AUPR	AUC	AUPR
DAESTB	0.8795	0.8745	0.6962	0.5270	0.6254	0.6302
GCNMMA	0.9208	0.8857	0.7210	0.5455	0.6167	0.6345
SubMDTA	0.8960	0.8624	0.6879	0.5363	0.4713	0.4778
GraphDTA	0.9223	0.8945	0.6992	0.5307	0.5797	0.5923
ML-DTI	0.9044	0.8687	0.6668	0.5494	0.5161	0.5183
**MGCNA**	**0.9462**	**0.9405**	**0.8537**	**0.6335**	**0.6849**	**0.6581**

### Case studies

To evaluate the capability of MGCNA in discovering novel drug-associated miRNAs through multi-source information integration, we conducted case studies using two common anticancer drugs, Docetaxel and Sorafenib. Specifically, for each target drug under investigation, we masked all its known miRNA associations by setting them as unknown. The training set comprised all remaining known MDAs in the dataset, along with an equal number of randomly selected unknown MDAs to maintain class balance. Following training, MGCNA generated prediction scores for all candidate miRNAs associated with the target drug. Finally, we screen the top 15 miRNAs in descending order based on their prediction scores and verify them against databases and published literature. The results are shown in Tables 4 and 5.

**Table 4:** The top-15 predicted Docetaxel-related miRNAs by MGCNA.

Rank	miRNA	Evidences	Rank	miRNA	Evidence
1	miR-29c-3p	ncRNADrug	11	miR-4443	PMID: 29151358
2	miR-27a-3p	ncRNADrug	12	miR-30a-5p	ncRNADrug
3	miR-196a-5p	ncRNADrug	13	miR-140-3p	PMID: 39348965
4	miR-671-5p	ncRNADrug	14	miR-221-5p	ncRNADrug
5	miR-100-5p	ncRNADrug	15	miR-18a-5p	Unconfirmed
6	miR-640	Unconfirmed			
7	miR-145-5p	ncRNADrug			
8	miR-490-3p	Unconfirmed			
9	miR-181b-5p	ncRNADrug			
10	miR-425-5p	Unconfirmed			

**Table 5:** The top-15 predicted Sorafenib-related miRNAs by MGCNA.

Rank	miRNA	Evidences	Rank	miRNA	Evidence
1	miR-181b-5p	ncRNADrug	11	miR-661	Unconfirmed
2	miR-196a-5p	ncRNADrug	12	miR-193b-5p	PMID: 25034398
3	miR-425-5p	ncRNADrug	13	miR-29a-3p	ncRNADrug
4	miR-34b-3p	ncRNADrug	14	miR-210-3p	ncRNADrug
5	miR-196a-3p	Unconfirmed	15	miR-558	Unconfirmed
6	miR-195-5p	ncRNADrug			
7	miR-361-3p	ncRNADrug			
8	miR-744-5p	ncRNADrug			
9	miR-383-5p	Unconfirmed			
10	miR-185-5p	Unconfirmed			

Docetaxel, a paclitaxel-based antitumor drug, is commonly used to treat breast, lung, and prostate cancers. Existing literature demonstrates that miRNAs contribute significantly to cancer cell resistance/sensitivity against common anticancer drugs and significantly influence Docetaxel resistance/sensitivity [29]. As shown in Table 4, 9 of the top 15 Docetaxel-related miRNAs predicted by MGCNA are confirmed through the ncRNADrug database. Furthermore, He et al. demonstrated that miR-4443 exhibited significant upregulation in drug-resistant cell lines through analysis of docetaxel-resistant breast cancer cells [30]. Specifically, Kwon et al. recently reported that miR-140-3p enhanced docetaxel sensitivity in lung adenocarcinoma by inhibiting PD-L1/ABCG2/MVP expression [31].

Sorafenib serves as a first-line chemotherapeutic agent for specific types of liver, kidney, and thyroid cancers. However, patients frequently develop resistance during treatment, resulting in diminished therapeutic efficacy [32]. To identify potentially relevant miRNAs, we analyzed Sorafenib using MGCNA (Table 5). 11 of the top 15 predicted miRNAs associated with Sorafenib were validated through the ncRNADrug database and literature evidence. Notably, Mao et al. demonstrated that restoration of miR-193b enhanced Sorafenib sensitivity in Hepatitis B virus-associated hepatocellular carcinoma [33]. The results of the two drug case studies indicate that MGCNA can reliably predict miRNAs associated with new drugs, underscoring its potential as a reliable tool for identifying miRNA-drug interactions.

## Discussion and conclusion

This study presents a novel multi-view fusion-based graph convolutional network framework with attention mechanism, MGCNA, for predicting miRNA-mediated drug resistance/sensitivity. The framework integrates domain knowledge and complex relationships of miRNAs and drugs to construct multiple views, including miRNA sequence view, drug structure view, gene-based miRNA/drug function view, and MDA-based miRNA/drug view. Subsequently, the framework employs a multi-view GCN encoder to learn representations of miRNAs and drugs from these diverse views, while an attention mechanism adaptively weights and integrates the most relevant information for accurate classification. Extensive experiments on manually curated datasets demonstrate that MGCNA achieves superior performance compared to all baseline methods, with model ablation studies confirming the value of each view. Case studies of Docetaxel and Sorafenib, supported by database and published literature, further confirm MGCNA’s capability to identify novel drug-related miRNAs through the integration of macro and micro of view information.

Despite the promising results, several limitations warrant consideration in future work. (1) The current MGCNA training dataset is constrained due to the sparse number of drugs available in ncRNADrug. In the future, we will implement transfer learning techniques to reduce the impact of insufficient drug data. (2) The current model only considers the existence of relationships between miRNAs and drugs, without distinguishing between drug resistance and sensitivity. In the future, more detailed interaction categories can be considered in construction MGCNA classification models. (3) Interpretability is a crucial research direction in bioinformatics. In future work, we will leverage conserved miRNA motifs and drug substructures to elucidate the molecular mechanisms underlying specific miRNA-drug resistance and sensitivity.

## Supporting information

S1 Text Overview of baseline bethods. (DOCX)
