# Supplementary material for: Multi-view fusion based on graph convolutional network with attention mechanism for predicting miRNA related to drugs
Source: PLoS Comput Biol. 2025 Nov 14;21(11):e1013703. doi: 10.1371/journal.pcbi.1013703 (PMC12617909; doi:10.1371/journal.pcbi.1013703)
Supplement: S1 Text — (DOCX) [file pcbi.1013703.s001.docx]

**1. Overview of Baseline Methods**

**DAESTB** is a deep learning method that employs an autoencoder to extract features for predicting small molecule-miRNA associations.

**GCNNMMA** utilizes graph neural networks and convolutional neural networks to extract drug structural features and miRNA sequence features, respectively, enabling the prediction of associations between miRNAs and small molecule drugs.

**SubMDTA** is a drug-target prediction method that applies GIN and BiLSTM to extract drug structure features and protein sequence features, respectively. In our implementation, miRNA sequences replace the original protein sequences as input.

**GraphDTA** is a drug-target prediction model that combines graph neural networks and traditional convolutional neural networks to extract drug structural features and protein sequence features respectively.

**ML-DTI** performs CNN to encode sequence information from drug SMILES and protein sequences, fully integrating their relationship through a mutual learning mechanism.

| Methods | Data information | Model parameters | Evaluation |
| --- | --- | --- | --- |
| DAESTB | Drug-gene association, drug structure, miRNA-gene association, miRNA-drug association | Deep autoencoder layers=4, learning rate=0.3 | 5-cv |
| GCNNMMA | Drug structure, miRNA sequence, miRNA-drug association | GNN layers=3, CNN layers=3 | 5-cv |
| SubMDTA | Drug structure, miRNA sequence, miRNA-drug association | GIN layers=3, n-gram=2,3,4, learning rate=0.0005 | 5-cv |
| GraphDTA | Drug structure, miRNA sequence, miRNA-drug association | GIN layers=5, learning rate=0.0005 | 5-cv |
| ML-DTI | Drug SMILES, miRNA sequence, miRNA-drug association | CNN layers=3, embedding=128 | 5-cv |
